# Comparative Analysis of Immune Activation Markers of CD8^+^ T Cells in Lymph Nodes of Different Origins in SIV-Infected Chinese Rhesus Macaques

**DOI:** 10.3389/fimmu.2016.00371

**Published:** 2016-09-21

**Authors:** Jinbiao Liu, Qianhao Xiao, Runhong Zhou, Yong Wang, Qiaoyang Xian, Tongcui Ma, Ke Zhuang, Li Zhou, Deyin Guo, Xu Wang, Wen-Zhe Ho, Jieliang Li

**Affiliations:** ^1^Animal Biosafety Level III Laboratory, Center for Animal Experiment, Wuhan University School of Basic Medical Sciences, Wuhan, China; ^2^Department of Pathology and Laboratory Medicine, Temple University Lewis Katz School of Medicine, Philadelphia, PA, USA

**Keywords:** simian immunodeficiency virus, immune activation, lymph nodes, CD4/CD8 ratio, CD8 activation

## Abstract

Altered T-cell homeostasis, such as expansion of CD8^+^ T cells to the secondary lymphatic compartments, has been suggested as a mechanism of HIV/simian immunodeficiency virus (SIV)-pathogenesis. However, the role of immune activation of CD8^+^ T cells in the CD4/CD8 turnover and viral replication in these tissues is not completely understood. In this study, we compared the expression of immune activation markers (CD69 and HLA-DR) on CD8^+^ T cells in the peripheral blood and lymph nodes (LNs) of SIV-infected/uninfected Chinese rhesus macaques. SIV-infected macaques had significantly higher percentages of CD8^+^CD69^+^ and CD8^+^HLA-DR^+^ T cells in all these anatomical compartments than uninfected macaques. LNs that located close to the gastrointestinal (GI) tract (colon, mesenteric, and iliac LNs) of SIV-infected macaques had profoundly lower numbers of CD4^+^ T cells, but no significant difference in expression of activation marker (CD8^+^CD69^+^ and CD8^+^HLA-DR^+^) as compared with the peripheral lymphatic tissues (axillary and inguinal LNs). The CD4/CD8 ratios were negatively correlated with the activation of CD8^+^ T cells in the overall LNs, with further associations with CD8^+^HLA-DR^+^ in GI LNs while CD8^+^CD69^+^ in peripheral LNs. These observations demonstrate that the increase of CD8^+^ T cell activation is a contributing factor for the decline of CD4/CD8 ratios in GI system.

## Introduction

Persistent immune activation is a hallmark of HIV infection in humans and simian immunodeficiency virus (SIV) infection in the non-natural host rhesus macaques (1–3). Microbial translocation at the gastrointestinal (GI) tract represents a major stimulus for immune activation that persists even after control of HIV replication (4, 5). The gut-associated lymphoid tissues (GALTs) are the primary sites of *in vivo* HIV infection and replication (6), as these lymphoid compartments contain the majority of CD4^+^ T cells in the body, the primary targets for HIV. Accumulating evidence indicate the GALTs play a key role in the persistence of HIV infection despite long-term antiretroviral therapy (7–9). The HIV-mediated immune activation has been extensively investigated in the peripheral system; however, the impact of immune activation on CD4^+^ T cell depletion in GI system remains elusive. In HIV disease, major injury to the gut immune system begins immediately after infection. There is an extensive depletion of CD4^+^ T memory cells in gut lymphoid tissues within 4–6 weeks of HIV infection (10). This immune dysregulation at the GALT mucosa is implicated in HIV enteropathy, accounting for approximately 60–80% of diarrhea in HIV-infected patients sometime during their illness, representing the most common clinical manifestation of AIDS (11).

Similar to HIV-infected subjects, SIV-infected macaques also commonly manifest diarrhea as AIDS complication (12). There is also a dramatic and selective depletion of CD4^+^ T cells predominately from the mucosal surface in GI tract (13, 14). A comparative analysis of cytotoxic T lymphocytes (CTLs) in the peripheral blood and lymph nodes (LNs) of SIV-infected rhesus monkeys by Kuroda et al. showed the phenotype similarity of SIV-specific CTL activity in these compartments (15). Gene expression profiling of gut mucosa and mesenteric LNs in SIV-infected macaques suggests that reduced immune activation and effective repair and regeneration of mucosal tissues correlate with long-term survival (16). However, due to the difference in distance to the gut mucosa, LNs at different tissue origins are not universal in the extent of immune response or CD4 loss during HIV/SIV infection. Unfortunately, thus far, there is no study to compare the immunological and virological responses between LNs of different location. In this study, we examined the different LNs from SIV-infected and -uninfected Chinese rhesus macaques, peripheral blood, jejunum, and colon intestines. We compared the profiles of CD4^+^ T cells, CD4/CD8 ratios, immune status of T cells, and inflammatory cytokines in these tissues. Our results show that a severe depletion of CD4^+^ T cells in GI LNs was accompanied by a profound CD8^+^ T cell immune activation and proinflammatory microenvironment, associated with higher viral load than that in peripheral LNs (pLNs).

## Materials and Methods

### Ethics Statement

All study protocols were approved by the Institutional Animal Care and Use Committee (IACUC) of the Wuhan University School of Medicine (Wuhan, China) in accordance with the regulations of the National Institute of Health “Guide for the Care and Use of Laboratory Animals” and all details of animal welfare and steps taken to ameliorate suffering were in accordance with the recommendations of the Weatherall report, “The use of non-human primates in research.” The animals were housed in an air-conditioned room with an ambient temperature of 16–26°C, a relative humidity of 40–70%, and a 12-h light–dark cycle at the Animal Bio-Safety Level-III (ABSL-III) laboratory of the Wuhan University School of Medicine, which were monitored in real time by a computer-based recording system. The ABSL-III Laboratory is certified by the Association for Assessment and Accreditation of Laboratory Animal Care International (AAALAC International). The animals were individually housed in stainless steel wire-bottomed cages with sufficient space (800 mm wide, 800 mm depth, and 1600 mm height) and provided with a commercial monkey diet. In addition to normal pellet food, fresh fruit was provided twice daily, and water was freely available at all times. The study animals were provided with an intellectually and physically enriched environment including rings, perches, forage boxes, puzzle feeders, music, and video in the room. Animal health was monitored daily by the animal care staff and veterinary personnel. Physiological parameters of the animal, such as heart rate, body temperature, and blood pressure, were monitored at constant intervals, and pain was evaluated by veterinarian. All experimental procedures were performed under anesthesia with intramuscular injection of ketamine hydrochloride (10 mg/kg) plus intramuscular injection of atropine (0.04 mg/kg), and all efforts were made to minimize suffering.

### Animals and Tissue Processing

Twelve Chinese rhesus macaques (RM; *Macaca mulatta*) were purchased from Sichuan Ping’an Non-Human Primates Breeding and Research Center (Sichuan Province, China). These animals (females, 5–6 years of age, 4–6 kg of weight) were individually housed and handled at the ABSL-III laboratory of the Wuhan University (Wuhan, China). The study animals were provided with an intellectually and physically enriched environment including rings, perches, forage boxes, and puzzle feeders. The animals were inoculated intravenously (i.v.) or intravaginally (i.vag.) with pathogenic R5 SIV strains (mac239, mac251, or macR71/17E). SIV-uninfected animals in the control group (*n* = 11) were from parallel studies. Collections and processing of blood were performed, as described previously (3, 17–19). Blood samples were used for routine flow cytometry analysis, and plasma was separated by centrifugation within 1 h of phlebotomy. All experimental procedures were performed under anesthesia with intramuscular injection of ketamine hydrochloride (10 mg/kg) and atropine (0.04 mg/kg), and all efforts were made to minimize suffering of the study animals. The humane endpoint criteria included the presentation of any of the following: (1) loss of 25% body weight from baseline weight when assigned to the protocol, (2) major organ failure or medical conditions unresponsive to treatment, and (3) tumors. If any one of these conditions was present, a humane endpoint was called and the animal was euthanized within 24 h. At necropsy, LNs from different tissues and the intestine tissues (Table 1) were collected from the animals after spontaneous death or euthanasia and subjected to lymphocyte isolation (20, 21). All the specimens were also cryopreserved or fixed in 10% buffered formalin.

**Table 1:** **Animals involved in this study and specimen information**.

Animal ID	SIV strain	Inoculation route	Times of necropsy^a^	Death complications	Plasma viral load^b^	CD4/CD8 ratio^c^	Lymph nodes																		
							Axillary	Inguinal	Salivary	Paratracheal	Adrenal	Carinal	Gastric	Duodenal	Jejunum	Iliac	Cecal	Colon	Mesentery	Celiac	Pelvic	IELs	Blood	Spleen	Plasma
WSN01	SIVmacR71/17E	i.v.	83 W	NeuroAIDS	3.48E + 07	0.18	√	√										√	√			√	√		√
WSN03	SIVmacR71/17E	i.v.	173 W	Diarrhea	7.16E + 07	1.61	√	√											√				√	√	√
WSN04	SIVmacR71/17E	i.v.	174 W	Diarrhea	2.03E + 06	0.17	√	√											√				√	√	√
WSP03	SIVmac251	i.v.	200 W	Diarrhea	2.97E + 07	0.50	√	√		√							√	√	√			√	√	√	√
WSB02	SIVmac239	i.v.	33 W	Unknown	2.36E + 09		√												√				√		√
WSL03	SIVmac239	i.v.	150 W	Diarrhea	9.64E + 07	0.07	√	√									√	√	√			√	√	√	√
WSL04	SIVmac239	i.v.	183 W	Diarrhea	2.86E + 07		√	√								√		√	√				√	√	√
WSL05	SIVmac239	i.v.	181 W	Diarrhea	7.35E + 07	1.39	√	√					√				√	√	√			√	√	√	√
WSP08	SIVmac239	i.v.	125 W	Diarrhea	8.34E + 08	0.12	√	√								√		√	√		√		√		√
WSP09	SIVmac239	i.v.	128 W	Tumor	4.65E + 07	0.5	√	√	√		√		√			√		√	√			√	√		√
WSP11	SIVmac239	i.vag.	149 W	Diarrhea	2.45E + 07	0.94	√	√		√		√	√	√		√		√	√	√		√	√	√	√
WSP12	SIVmac239	i.vag.	216 W	Diarrhea	4.02E + 07	0.7	√	√											√				√	√	√
HK01	None	None				0.91	√	√										√					√		
WSE01	None	None				1.74	√	√	√						√	√	√	√	√			√	√	√	
T096041	None	None				2.03	√	√									√	√	√			√	√	√	
T096057	None	None				1.15	√	√									√	√	√			√	√	√	
T106027	None	None				0.82	√	√										√	√			√	√	√	
10341	None	None				1.05	√	√										√	√			√	√	√	
10025	None	None				1.27	√	√										√	√			√	√	√	
10409	None	None				0.81	√	√										√	√			√	√	√	
10013	None	None				1.00	√	√										√	√			√	√	√	
10045	None	None				0.96	√	√										√	√			√	√	√	
10343	None	None				0.82	√	√										√	√			√	√	√	

*^a^Date are weeks relative to the time of SIV infection*.

*^b^Viral load at necropsy*.

*^c^CD4/CD8 ratio in whole blood*.

*IELs, intraepithelial lymphocytes*.

### Lymphocyte Isolation from LNs and Intestines

Lymph node-derived lymphocytes were mechanically homogenized and passed through a 70-μm cell strainer to remove residual tissue fragments. These LNs were classified into pLNs or GI LNs based on their locations. The pLNs include LNs from the fossa axillaris and inguen where there were available in the SIV-infected animals. The GI LNs include those LNs from the colon, mesentery, and ileum that were closer to the GI tract. Some animals might have several LNs from the same tissue, while some might have no particular LNs (Table 1). To isolate lymphocytes from colon and jejunum intestines, these tissues were cut into small pieces and digested with 1 mg/ml collagenase for 1 h at 37°C with constant shaking, and then passed through a 70-μm cell strainer. Intestinal intraepithelial lymphocytes (IELs) were separated by 30–60% Percoll-plaque density gradient centrifugation, as described previously (22, 23). All samples were processed, cryopreserved, fixed (1% paraformaldehyde), and analyzed within 24 h of collection.

### Flow Cytometric Analysis

Six-parameter flow cytometric analysis was performed on whole blood, IELs, and T cells from LNs according to standard procedures (24) using a panel of monoclonal antibodies (mAbs) that were originally designed to detect human molecules but that we and others have shown to be cross-reactive with rhesus monkey (3, 19, 25, 26). The antibodies used were as follows: anti-CD3-FITC (clone SP34), anti-CD4-APC-H7 (clone L200), anti-CD8-PE-Cy7 (clone RPA-T8), anti-CD69-PE (clone FN50), and anti-HLA-DR-PerCP-Cy5.5 (clone G46-6) (all from BD Pharmingen). Isotype antibody was used for negative control of CD69 and HLA-DR expression. Flow cytometric acquisition and analysis of samples were performed on at least 100,000 events on a BD Verse cytometer driven by the FACS Verse software (BD Biosciences). Analysis of the acquired data was performed using FlowJo software (TreeStar, Ashland, OR, USA). The CD4^+^ and CD8^+^ T cell percentages were based on the CD3^+^ T cells, and the activation markers on CD4^+^ and CD8^+^ T cells were based on CD4^+^CD3^+^ and CD8^+^CD3^+^ T cells, respectively.

### Quantitative Real-time Reverse Transcription PCR

Plasma RNA were extracted and reverse transcribed into cDNA, and real-time RT-PCR for SIV gag were performed, as described previously (3). Briefly, SIV RNA copies per milliliter of plasma were determined by comparing sample cycle threshold (Ct) to that of a known standard of SIV gag RNA. Duplicate samples were analyzed, and the limit of detection was 200 SIV RNA copies per milliliter plasma. Real-time PCR to quantify tissue-associated viral loads was also performed on LNs or intestine tissues from animals as shown in Table 1. RNA was extracted from cryopreserved tissues, and 2 μg of RNA was reverse transcribed into cDNA for real-time RT-PCR measurement of SIV gag and host cytokines/chemokines [interleukin (IL)-10, C–C motif ligand 2 (CCL2), interferon (IFN)-γ, and tumor necrosis factor alpha (TNF-α)] using iQ SYBR Green Supermix (Bio-Rad Laboratories, Hercules, CA, USA). The levels of glyceraldehyde-3-phosphate dehydrogenase (GAPDH) mRNA were used as an endogenous reference to normalize the quantities of mRNAs.

### Statistical Analysis

Data are presented as mean ± SD per group as uninfected or SIV-infected monkeys or as pLNs or GI LNs. These data were statistically analyzed using GraphPad Prism software (La Jolla, CA, USA). Data between two groups were compared by a non-parametric Mann–Whitney *T* test, and *p* < 0.05 was considered as significant.

## Results

### Viral Load and CD4/CD8 Ratios in SIV-Infected Animals

**Figure 1:**
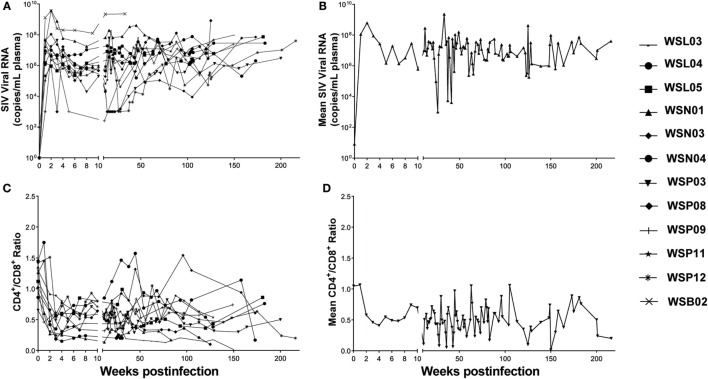
**Plasma SIV load and CD4/CD8 ratio in SIV-infected Chinese rhesus macaques**. Twelve animals were i.v. or i.vag. inoculated with different strains of SIV as indicated in Table 1. **(A)** The plasma viral load in individual animal was monitored. **(B)** Mean values of plasma viral load in the SIV-infected macaques. **(C)** CD4/CD8 ratio in individual animal was monitored. **(D)** Mean values of CD4/CD8 ratios in the SIV-infected macaques.

We initially infected eight animals with SIV_mac239_ and collected LNs from different lymphoid tissues at necropsy with six out of eight animals associated with diarrhea AIDS complication at the end-stage of infection. However, due to the limited numbers of LNs available from these animals, we included the specimens from one animal infected with SIV_mac251_ and three animal infected with SIV_macR71/17E_. The viral loads in the plasma were monitored periodically. All the animals became infected as evidenced by increased levels of plasma SIV gag RNA (Figure 1A). The CD4/CD8 ratio decreased significantly in these animals 2 weeks after SIV infection (Figure 1C). The viral loads and CD4/CD8 ratios tend to change in opposite directions during the course of infection (Figures 1B,D).

### Decline of CD4^+^ T Cells in Different Tissues of SIV-Infected Animals

**Figure 2:**
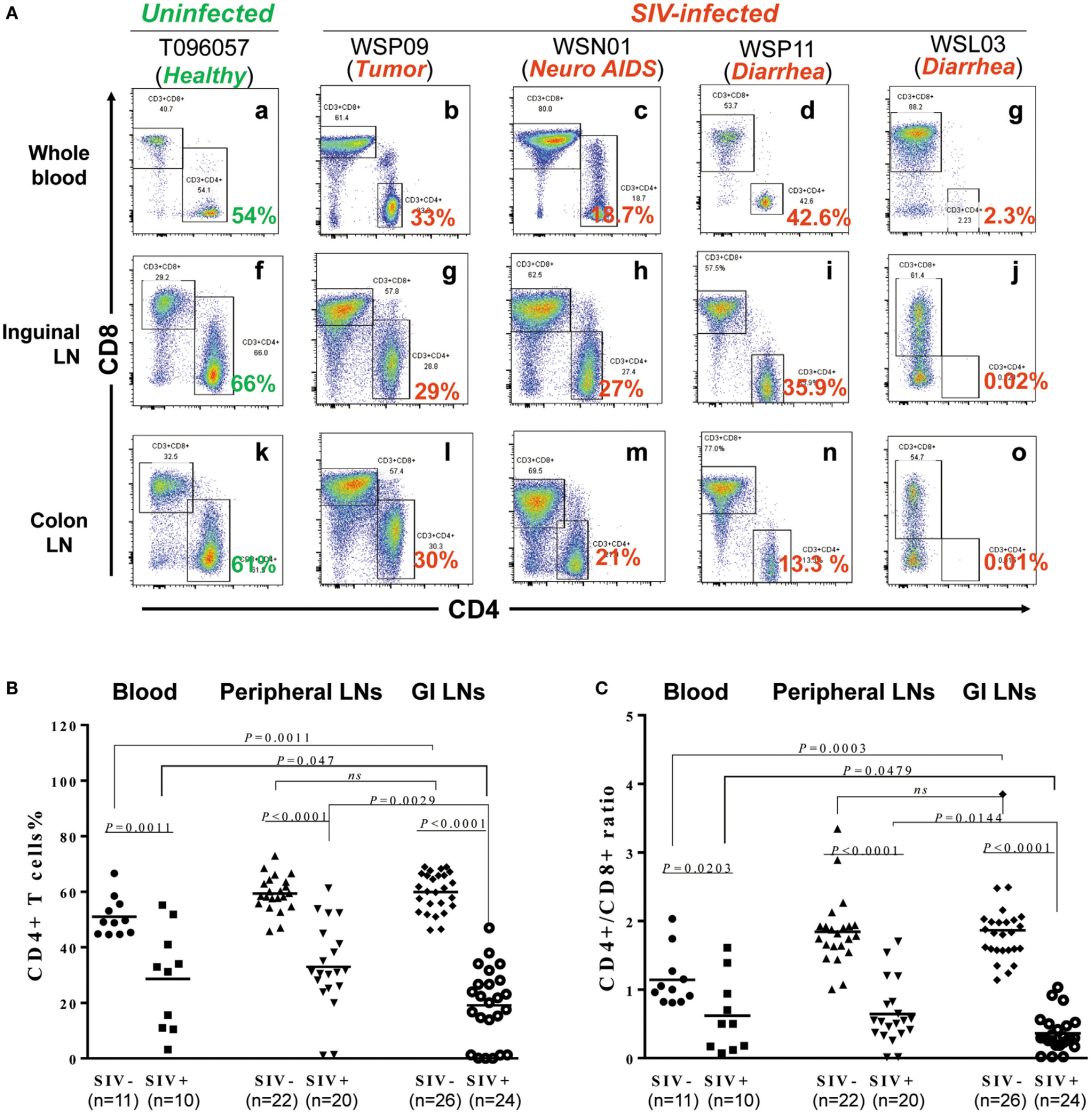
**Effect of SIV infection on the depletion of CD4^+^ T cells in different tissues**. **(A)** CD4^+^ T cell percentages in the blood, inguinal LN, and colon LN of one uninfected macaques (T096057) and four SIV-infected macaques (WSP09, WSN01, WSP11, and WSL03). **(B)** Statistical analysis of CD4^+^ T cell percentages in the blood, peripheral LNs, and GI LNs of SIV-infected and -uninfected macaques. **(C)** Statistical analysis of CD4/CD8 ratio in the blood, peripheral LNs, and GI LNs of SIV-infected and -uninfected macaques. *N* means the number of specimens. The “*n*” represents the number of specimens from all monkeys involved in this study.

We next examined the extent of CD4^+^ T cell decline in lymphoid tissues of SIV-infected macaques. Figure 2A shows the CD4^+^ T cell percentages in the peripheral blood, inguinal LNs, and colon from one uninfected animal (T096057) and four SIV-infected animals (WSP09, WSN01, WSP11, and WSL03). CD4^+^ T cells were dominant (54–66%) in all these lymphoid tissues from the uninfected macaque. In contrast, SIV-infected animals had much lower percentages of CD4^+^ T cells in these compartments. We next analyzed whether there was significant difference between the two groups in the CD4^+^ T cell abundance in these tissues. As shown in Figure 2B, the CD4^+^ T cell percentages differed all significantly in these compartments between the SIV-infected and -uninfected animals (Figure 2B; *p* < 0.05). Furthermore, in SIV-infected animals, the depletion of CD4^+^ T cells was more profound in the GI LNs as compared with those in blood (*p* = 0.047) and pLNs (*p* = 0.0029). In contrast, in the uninfected animals, the CD4^+^ T cell percentages in GI LNs were comparable to those in pLNs. Consistent with the difference in CD4^+^ T cell percentages between the two groups, SIV-infected animals had significant lower CD4/CD8 ratios than uninfected animals in all the corresponding compartments (*p* < 0.05; Figure 2C). In addition, in uninfected animals, the mean CD4/CD8 ratio in GI LNs was 1.82 (1.34–2.49), which was comparable to that in the pLNs (mean 1.76; 1.54–1.94; *p* = 0.60) but was significantly higher than that in the blood (mean 1.24; 0.81–2.03; *p* = 0.0003). On the contrary, in the SIV-infected monkeys, the mean CD4/CD8 ratio in GI LNs was 0.36 (0.02–1.03), which was significantly lower than those in both blood (mean 0.62, 0.18–1.61, *p* = 0.0479) and pLNs (mean 0.64, 0.014–1.70, *p* = 0.0144).

### CD4^+^ T Cell Depletion in IELs

**Figure 3:**
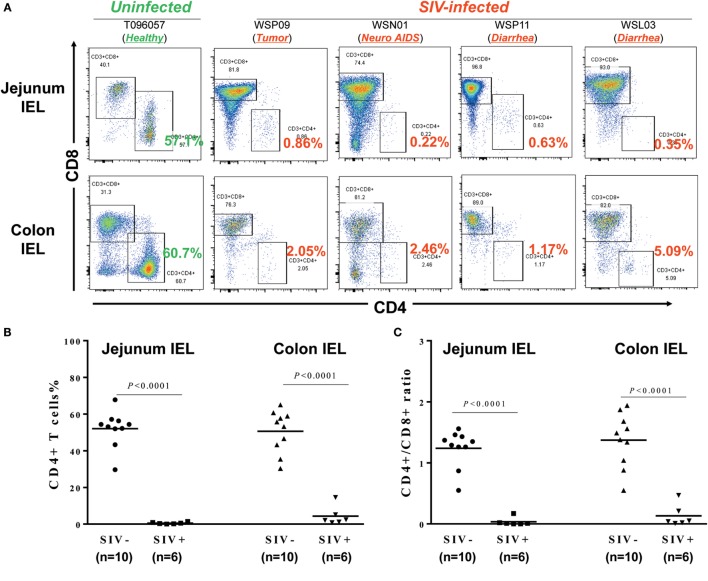
**Effect of SIV infection on the depletion of CD4^+^ T cells in the intraepithelial lymphocytes (IELs)**. **(A)** Flow cytometric analysis of CD4^+^ and CD8^+^ T cells in IEL from one uninfected and four SIV-infected macaques. Approximately 6–10 cm long pieces of jejunum and colon were collected from SIV-infected or -uninfected macaques at necropsy. **(B)** Statistical analysis of CD4^+^ T cell percentages in jejunum and colon IEL of SIV-infected (*N* = 6) and -uninfected macaques (*N* = 10). **(C)** Statistical analysis of CD4/CD8 ratio in jejunum and colon IEL of SIV-infected (*N* = 6) and -uninfected macaques (*N* = 10).

Intraepithelial lymphocytes are lymphocytes in the epithelial layer of mammalian mucosal linings, such as the GI tract. IELs have been reported to be primary targets for HIV acquisition (27). We thus isolated the IELs from different parts of the intestines from SIV-infected and -uninfected macaques. Figure 3A shows the CD4^+^ T cell percentages in IELs from 1 uninfected macaque (T096057; 1 out of 10 animals from which IELs were obtained) and four SIV-infected macaques (WSP09, WSN01, WSP11, and WSL03; 4 out of 6 animals from which IELs were obtained). It was found that in the uninfected macaque (T096057), the jejunum and colon IELs contained 57.1 and 60.7% of CD4^+^ T cells, respectively. In contrast, the CD4^+^ T cells in the IELs from the intestinal tissues of the four SIV-infected macaques were almost depleted (<1% in the jejunum IELs and <5% in the colon IELs). The percentages of CD4^+^ T cells (Figure 3B) and CD4/CD8 ratio (Figure 3C) in IELs from SIV-infected animals were significantly lower than those in IELs from uninfected animals.

### T Cell Activation of SIV-Infected Monkeys

**Figure 4:**
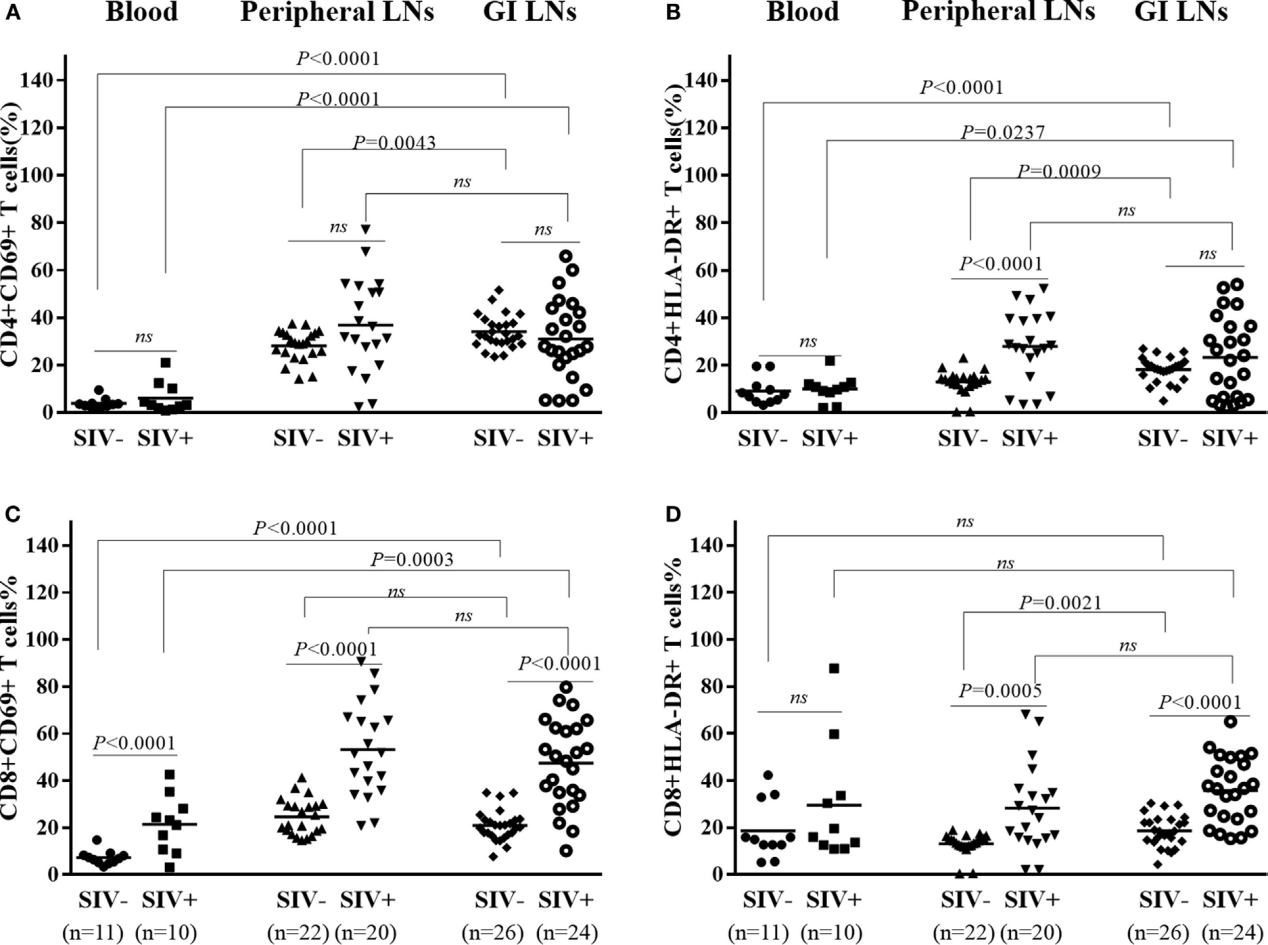
**T cells activation in different tissues of SIV-infected and -uninfected rhesus macaques**. Statistical analysis of activation marker CD69 and HLA-DR expression on CD4^+^ **(A,B)** and CD8^+^ **(C,D)** T cells in different tissues of SIV end-staged infected and uninfected rhesus macaques. *N* means the number of specimens.

Hyperactivation of T cells, particularly CD8^+^ T cells, in the peripheral blood is a hallmark of chronic HIV/SIV infection. However, the activation status of T cells in the LNs of different origins remains unclear. We thus investigated the T cell activation in the different tissues in both SIV-infected and -uninfected animals. As shown in Figures 4A,B, there was no significant difference in the percentages CD4^+^CD69^+^ and CD4^+^HLA-DR^+^ in the three types of lymphoid tissues of the uninfected animals as compared with SIV-infected animals, except for a higher percentage of CD4^+^HLA-DR^+^ in the pLNs of SIV-infected animals than that of uninfected animals (*p* < 0.0001). On the contrary, the percentages CD8^+^CD69^+^ and CD8^+^HLA-DR^+^ in these tissues of the infected animals were all significantly higher as compared with those of uninfected animals, except for no difference in the peripheral blood CD8^+^HLA-DR^+^ proportions between the infected and uninfected animals (Figures 4C,D).

### Correlation of Activated T Cells with CD4/CD8 Ratio in SIV-Infected Monkeys

**Figure 5:**
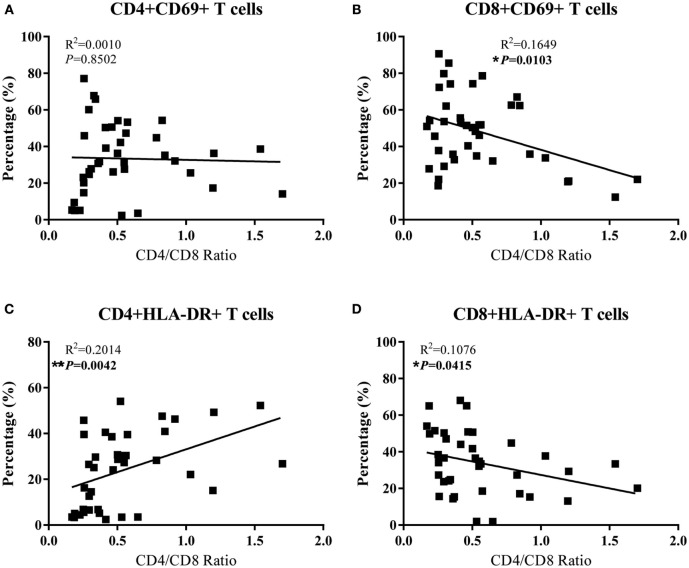
**Correlation analysis of the activation of CD4^+^ T cells and CD8^+^ T cells with CD4/CD8 ratio in LNs of SIV-infected monkeys**. **(A)** The percentage of CD4^+^CD69^+^ had no association with CD4/CD8 ratio. **(B)** The percentage of CD8^+^CD69^+^ was negatively correlated with CD4/CD8 ratio. **(C)** The percentage of CD4^+^HLA-DR^+^ was positively associated with CD4/CD8 ratio. **(D)** The percentage of CD8^+^HLA-DR^+^ was negatively correlated with CD4/CD8 ratio.

To determine the contribution of T cells activation on CD4 depletion, we examined whether there is an association between the levels of T cell activation and CD4/CD8 ratios in the peripheral and GI LNs from SIV-infected monkeys. As shown in Figures 5B,D, the CD4/CD8 ratio of these LNs was negatively correlated with CD8^+^ T cell activation in terms of the percentages of CD8^+^CD69^+^ T cells (*p* = 0.0103) and CD8^+^HLA-DR^+^ T cells (*p* = 0.0415) in these tissues. In contrast, CD4^+^ T cell activation in terms of the percentage of CD4^+^HLA-DR^+^ T cells was positively correlated with the tissue CD4/CD8 ratio (Figure 5C, *p* = 0.0042). There was no correlation between CD4/CD8 ratio and the level of CD4^+^CD69^+^ (Figure 5A). Further analysis shows that in pLNs, CD4/CD8 ratio was inversely correlated with CD8^+^CD69^+^ T cell percentage (*p* = 0.0033), whereas in GI LNs, it was inversely correlated with CD8^+^HLA-DR^+^ T cell percentage (*p* = 0.0482) (Figure S1 in Supplementary Material).

### SIV Loads and Cytokine Profiles in LNs

**Figure 6:**
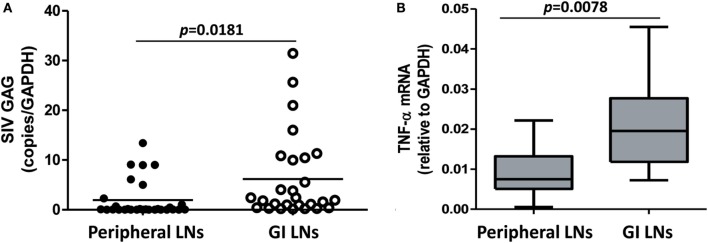
**SIV load and TNF-α level in the peripheral LNs and GI LNs of SIV-infected monkeys**. The LNs were collected from monkeys at necropsy, and total RNA was extracted. The expression of SIV gag **(A)** and TNF-α **(B)** was measured by quantitative RT-PCR. These data were normalized to the expression of GAPDH.

In HIV infection, LNs are important sites of viral replication, in which the high local density of CD4^+^ T cells and other target cells may favor cell contact-mediated viral spread (28). We next examined the SIV loads and the cytokine expression in the different LNs of SIV-infected monkeys. As shown in Figure 6A, the defined GI LNs had significantly higher viral loads (*p* = 0.0181) than LNs from peripheral tissues (mainly inguinal and axillary LNs). The expression of TNF-α was also significantly higher in GI LNs than that in pLNs (*p* = 0.0078; Figure 6B). However, there was no significant difference in the expression levels of CCL2, IL-1β, and IL-8 between the two types of LNs (data not shown).

## Discussion

In this study, we examined the T-cell homeostasis and cytokine profiles in the peripheral blood and LNs from SIV-infected Chinese rhesus macaques. Approximately 80% of the SIV-infected macaques developed AIDS-related diarrhea at the end-stage of infection. We found that SIV infection significantly reduced the proportion of CD4^+^ T cells and CD4/CD8 ratios in both the peripheral blood and LNs. In addition, LNs from the GI lymphatic tissues (colon LN, mesenteric LN, and iliac LN) had more severe loss of CD4^+^ T cells than those from the peripheral lymphatic tissues (e.g., axillary and inguinal LNs). The most severe impairment of T-cell homeostasis occurs at the intestinal mucosa, as evidenced by a severe depletion of CD4^+^ T cells in the IELs isolated from colon and jejunum.

The GALTs constitute the largest immune compartment of the body and have a large mucosal surface in close proximity to the external environment (29, 30). It is estimated that T cells associated with the small intestinal epithelium alone may account for more than 60% of the total body lymphocytes (31–33). Previous studies have reported the preferential impairment of the intestinal mucosal immune system by HIV/SIV infection (8, 34–37). Our results show that the GALTs, including GI LNs, have more severe declines in both CD4^+^ T cell percentages and the CD4/CD8 ratios than in peripheral blood at the end-stage of infection. Indeed, a striking depletion of intestinal CD4^+^ T cells was noted in macaques within days of SIV infection, at a time when little or no CD4^+^ T cell depletion was evident in the peripheral blood, mesenteric, and axillary LNs (29). The depletion of CD4^+^ T cells in the GI tract occurred coincidently with productive infection of large numbers of mononuclear cells at this site. Massive infection caused depletion of memory CD4^+^ T cells has also been revealed during acute SIV infection (14). Specifically, 30–60% of CD4^+^ memory T cells throughout the body were infected by SIV at the peak of infection, and most of these infected cells disappeared with 4 days. The severe depletion of CD4^+^CD8^−^ single-positive T cells and CD4^+^CD8^+^ double-positive T cells in intestinal lamina propria lymphocytes (LPLs) and IELs was found during primary SIV infection and persisted throughout the entire course (38). There was no restoration of these cells in intestinal mucosa in the asymptomatic or terminal stage of SIV infection (38). Even with antiretroviral therapy intervention, the mucosal CD4^+^ T cell restoration was slower and less significant than that of cells in the peripheral blood (39).

Chronic immune activation is considered as the major driving force of CD4^+^ T-cell depletion and disease progression of HIV/SIV infection. Hyperactivation of T cells, particularly CD8^+^ T cells, in the peripheral blood is a hallmark of chronic HIV/SIV infection (40). GI complications in HIV/SIV infection are indicative of impaired intestinal mucosal immune system. However, the activation status of T cells in the LNs derived from the GI tissues with comparison to those from peripheral tissues remains unclear. Our data show that SIV-infected animals had significantly upregulated CD69 and HLA-DR expression of CD8^+^ T cells in peripheral blood and LNs from both the GI and peripheral tissues than uninfected animals (Figure 4). In addition, we also demonstrated that there was no significantly difference in the expression of activation markers between GI LN and pLN in SIV-infected monkeys, despite the more profound CD4 depletion and decline of CD4/CD8 ratio in the GI LN compared with the pLN (Figure 2). Overall, the CD4/CD8 ratio was negatively correlated with CD8^+^ T cell activation in terms of the both CD69^+^ (*p* = 0.0103) and HLA-DR^+^ T cells (*p* = 0.0415) of these LNs. However, as compared with the negative correlation with the increased CD69 expression in CD8^+^ T cells in the pLNs, the CD4/CD8 ratio was inversely correlated with the increased HLA-DR expression of CD8^+^ T cells in the GI LNs. The infiltration of CD8^+^ T cells to the GI lymphatic compartments has been implicated in the CD4^+^ T cell loss during HIV/SIV infection. Increased lymphocytic infiltration of intestinal tissues has been observed in patients infected with HIV and in SIV-infected macaques with resultant imbalance of CD4:CD8 T cell ratio (41–43). This signifies the dysregulation of immune hemostasis at the intestinal tissues in AIDS-related GI disease, in particular diarrhea. In our study, we observed that there was no significant difference in the percentages of CD4^+^CD69^+^ and CD4^+^HLA-DR^+^ in the three types of lymphoid tissues of the uninfected animals as compared with SIV-infected animals, except for a higher percentage of CD4^+^HLA-DR^+^ in the pLNs of SIV-infected animals at necropsy. On the contrary, in these chronic SIV-infected macaques before the development of AIDS-related diarrhea, both CD4^+^ T cells and CD8^+^ T cells expressed higher immune activation markers CD69 and HLA-DR in the peripheral blood (Figure S1 in Supplementary Material). Thus, the severe depletion of CD4^+^ T cells at the end-stage of SIV infection might contribute to the diminished significance of CD4^+^ T cell activation (39).

In HIV infection, LNs are important sites of viral replication, as they contain the high density of CD4^+^ T cells and other target cells for the virus (28). The significance of pLNs in the pathogenesis of HIV/SIV infection has been extensively studied (44–46). However, dynamic changes of T lymphocytes in pLNs or blood were not adequately represented intestinal tissues (10). Thus far, there is very limited information about the difference between the viral loads and cytokine profiles in LNs of different origin during HIV or SIV infection. Anatomically, LNs derived from different tissues drain lymph vessels regionally. For example, axillary LNs drain lymph vessels from breast and chest and are clinically significant in breast cancer diagnosis (47). On the contrary, mesenteric LNs drain the GI mucosa tissue (48) and are thought as the crossroad of systemic and mucosal immunity within which lymphoid cells from both interact and T cell maturation continues (49, 50). As such, LNs close to the GI tract drain intestinal tissues that are regularly exposed to a complex and diverse assortment of antigens from both microbial and dietary sources (51, 52). We showed that the GI LNs had higher viral load and TNF-α expression than pLNs. A recent study by Orenstein (53) examined the HIV RNA and p24 expression in GALT and deep LNs acquired during surgery on HIV-infected patients. It was found that the level of viral expression in the deep LNs, e.g., mesenteric and retroperitoneal, was at least equivalent to that seen in superficial LNs (i.e., inguinal, axillary, and cervical), tonsils, and adenoids. It is known that the translocating microbes and microbial products are phagocytosed first within the lamina propria and the mesenteric LNs during viral infection prior to dissemination to peripheral blood and LNs (54). Therefore, mesenteric LNs associated with CD8^+^ T cell activation play a major role in virus dissemination to the peripheral circulation after HIV infection. Indeed, mesenteric LNs have been reported as major cellular reservoirs that cause rebound of plasma viremia upon cessation of therapy (55), which may be attributable to the lack of viral control because of apoptotic death of CD8^+^ T cells (56, 57). Altered T-cell homeostasis, such as infiltration of CD8^+^ T cells to the lymphatic compartments in intestinal mucosa, has been indicated as a mechanism of HIV/SIV-associated enteropathy (41–43). We showed that GI LNs had higher level of activated CD8^+^ T cell and TNF-α than pLNs. In addition, there was a negative correlation between the depletion of CD4^+^ T cells and viral loads in the GI LNs, but not in the pLNs (data not shown). This finding provide a mechanism for the viral-mediated destruction of T cell hemostasis, rather than immune activation-mediated cell turnover in GI LNs (58). Therefore, the GI LNs, particularly the mesenteric LNs, are of great significance in evaluating the mucosal and systemic immune activation in addition to the IELs and peripheral blood.

In summary, we showed the significant depletion of CD4^+^ T cells and decline of CD4/CD8 ratios in peripheral and GI tract lymphatic tissues particularly in IELs in the SIV-infected macaques with AIDS-associated diarrhea. The CD4/CD8 ratio in GI LNs was negatively correlated with CD8^+^HLA-DR^+^ T cell percentage while with CD8^+^CD69^+^ T cell in pLNs. Compared with pLNs, GI LNs exhibited higher SIV load and TNF-α level. These observations demonstrated that CD8^+^ T cell activation, particularly the increased HLA-DR^+^ expression and the local inflammatory microenvironment, contribute to HIV-associated GI disease.

## Author Contributions

J. Liu, J. Li, and W-ZH conceived and designed the experiments. J. Liu, J. Li, R. Zhou, Q. Xiao, Y. Wang, Q. Xian, T. Ma, K. Zhuang, and L. Zhou performed the experiments. J. Liu, J. Li, and W-ZH analyzed the data. XW and DG contributed reagents/materials/analysis tools. J. Liu, J. Li, and W-ZH wrote the paper.

## Conflict of Interest Statement

The authors declare that the research was conducted in the absence of any commercial or financial relationships that could be construed as a potential conflict of interest.

## References

[1] Brenchley JM Price DA Schacker TW Asher TE Silvestri G Rao S Microbial translocation is a cause of systemic immune activation in chronic HIV infection. Nat Med (2006) 12:1365–71. 10.1038/nm1511 17115046

[2] Pandrea I Gaufin T Brenchley JM Gautam R Monjure C Gautam A Cutting edge: experimentally induced immune activation in natural hosts of simian immunodeficiency virus induces significant increases in viral replication and CD4+ T cell depletion. J Immunol (2008) 181:6687–91. 10.4049/jimmunol.181.10.6687 18981083 PMC2695139

[3] Bao R Zhuang K Liu J Wu J Li J Wang X Lipopolysaccharide induces immune activation and SIV replication in rhesus macaques of Chinese origin. PLoS One (2014) 9:e98636. 10.1371/journal.pone.0098636 24918575 PMC4053387

[4] Klatt NR Funderburg NT Brenchley JM. Microbial translocation, immune activation, and HIV disease. Trends Microbiol (2013) 21:6–13. 10.1016/j.tim.2012.09.001 23062765 PMC3534808

[5] Marchetti G Tincati C Silvestri G. Microbial translocation in the pathogenesis of HIV infection and AIDS. Clin Microbiol Rev (2013) 26:2–18. 10.1128/CMR.00050-12 23297256 PMC3553668

[6] Kraehenbuhl JP The gut-associated lymphoid tissue: a major site of HIV replication and CD4 cell loss. Trends Microbiol (1998) 6:419–20. 10.1016/S0966-842X(98)01393-6 9846350

[7] Nilsson J Kinloch-De-Loes S Granath A Sonnerborg A Goh LE Andersson J. Early immune activation in gut-associated and peripheral lymphoid tissue during acute HIV infection. AIDS (2007) 21:565–74. 10.1097/QAD.0b013e3280117204 17314518

[8] Chun TW Nickle DC Justement JS Meyers JH Roby G Hallahan CW Persistence of HIV in gut-associated lymphoid tissue despite long-term antiretroviral therapy. J Infect Dis (2008) 197:714–20. 10.1086/527324 18260759

[9] Tincati C Biasin M Bandera A Violin M Marchetti G Piacentini L Early initiation of highly active antiretroviral therapy fails to reverse immunovirological abnormalities in gut-associated lymphoid tissue induced by acute HIV infection. Antivir Ther (2009) 14:321–30. 19474466

[10] Brenchley JM Schacker TW Ruff LE Price DA Taylor JH Beilman GJ CD4+ T cell depletion during all stages of HIV disease occurs predominantly in the gastrointestinal tract. J Exp Med (2004) 200:749–59. 10.1084/jem.20040874 15365096 PMC2211962

[11] Zacharof AK AIDS-related diarrhea pathogenesis, evaluation and treatment. Ann Gastroenterol (2001) 14:22–6.

[12] Kewenig S Schneider T Hohloch K Lampe-Dreyer K Ullrich R Stolte N Rapid mucosal CD4(+) T-cell depletion and enteropathy in simian immunodeficiency virus-infected rhesus macaques. Gastroenterology (1999) 116:1115–23. 10.1016/S0016-5085(99)70014-4 10220503

[13] Picker LJ Hagen SI Lum R Reed-Inderbitzin EF Daly LM Sylwester AW Insufficient production and tissue delivery of CD4+ memory T cells in rapidly progressive simian immunodeficiency virus infection. J Exp Med (2004) 200:1299–314. 10.1084/jem.20041049 15545355 PMC2211921

[14] Mattapallil JJ Douek DC Hill B Nishimura Y Martin M Roederer M. Massive infection and loss of memory CD4+ T cells in multiple tissues during acute SIV infection. Nature (2005) 434:1093–7. 10.1038/nature03501 15793563

[15] Kuroda MJ Schmitz JE Charini WA Nickerson CE Lord CI Forman MA Comparative analysis of cytotoxic T lymphocytes in lymph nodes and peripheral blood of simian immunodeficiency virus-infected rhesus monkeys. J Virol (1999) 73:1573–9. 9882363 10.1128/jvi.73.2.1573-1579.1999 PMC103982

[16] George MD Verhoeven D Mcbride Z Dandekar S. Gene expression profiling of gut mucosa and mesenteric lymph nodes in simian immunodeficiency virus-infected macaques with divergent disease course. J Med Primatol (2006) 35:261–9. 10.1111/j.1600-0684.2006.00180.x 16872289

[17] Sumpter B Dunham R Gordon S Engram J Hennessy M Kinter A Correlates of preserved CD4(+) T cell homeostasis during natural, nonpathogenic simian immunodeficiency virus infection of sooty mangabeys: implications for AIDS pathogenesis. J Immunol (2007) 178:1680–91. 10.4049/jimmunol.178.3.1680 17237418

[18] Brenchley JM Paiardini M Knox KS Asher AI Cervasi B Asher TE Differential Th17 CD4 T-cell depletion in pathogenic and nonpathogenic lentiviral infections. Blood (2008) 112:2826–35. 10.1182/blood-2008-05-159301 18664624 PMC2556618

[19] Engram JC Cervasi B Borghans JA Klatt NR Gordon SN Chahroudi A Lineage-specific T-cell reconstitution following in vivo CD4+ and CD8+ lymphocyte depletion in nonhuman primates. Blood (2010) 116:748–58. 10.1182/blood-2010-01-263814 20484087 PMC2918331

[20] Gabrilovich D. Isolation of dendritic cells from mouse lymph nodes. Methods Mol Med (2001) 64:3–7. 10.1385/1-59259-150-7:3 21374244

[21] Fletcher AL Malhotra D Acton SE Lukacs-Kornek V Bellemare-Pelletier A Curry M Reproducible isolation of lymph node stromal cells reveals site-dependent differences in fibroblastic reticular cells. Front Immunol (2011) 2:35. 10.3389/fimmu.2011.00035 22566825 PMC3342056

[22] Montufar-Solis D Klein JR. An improved method for isolating intraepithelial lymphocytes (IELs) from the murine small intestine with consistently high purity. J Immunol Methods (2006) 308:251–4. 10.1016/j.jim.2005.10.008 16337223

[23] Pan D Das A Liu D Veazey RS Pahar B. Isolation and characterization of intestinal epithelial cells from normal and SIV-infected rhesus macaques. PLoS One (2012) 7:e30247. 10.1371/journal.pone.0030247 22291924 PMC3266894

[24] Silvestri G Sodora DL Koup RA Paiardini M O’Neil SP Mcclure HM Nonpathogenic SIV infection of sooty mangabeys is characterized by limited bystander immunopathology despite chronic high-level viremia. Immunity (2003) 18:441–52. 10.1016/S1074-7613(03)00060-8 12648460

[25] Paiardini M Cervasi B Reyes-Aviles E Micci L Ortiz AM Chahroudi A Low levels of SIV infection in sooty mangabey central memory CD(4)(+) T cells are associated with limited CCR5 expression. Nat Med (2011) 17:830–6. 10.1038/nm.2395 21706028 PMC3253129

[26] Pallikkuth S Micci L Ende ZS Iriele RI Cervasi B Lawson B Maintenance of intestinal Th17 cells and reduced microbial translocation in SIV-infected rhesus macaques treated with interleukin (IL)-21. PLoS Pathog (2013) 9:e1003471. 10.1371/journal.ppat.1003471 23853592 PMC3701718

[27] Swaims A Haaland R Lupo D Evans-Strickfaden T Kohlmeier J Haddad L Intra-epithelial T lymphocytes from the cervical-vaginal mucosa of healthy women contain a majority population of CD4+ cells and express high levels of the HIV-1 co-receptor CCR5. J Immunol (2013) 190(1 Suppl):210–2.

[28] Murooka TT Deruaz M Marangoni F Vrbanac VD Seung E Von Andrian UH HIV-infected T cells are migratory vehicles for viral dissemination. Nature (2012) 490:283–7. 10.1038/nature11398 22854780 PMC3470742

[29] Veazey RS Demaria M Chalifoux LV Shvetz DE Pauley DR Knight HL Gastrointestinal tract as a major site of CD4+ T cell depletion and viral replication in SIV infection. Science (1998) 280:427–31. 10.1126/science.280.5362.427 9545219

[30] Koboziev I Karlsson F Grisham MB. Gut-associated lymphoid tissue, T cell trafficking, and chronic intestinal inflammation. Ann N Y Acad Sci (2010) 1207(Suppl 1):E86–93. 10.1111/j.1749-6632.2010.05711.x 20961311 PMC3075575

[31] Guy-Grand D Vassalli P. Gut intraepithelial T lymphocytes. Curr Opin Immunol (1993) 5:247–52. 10.1016/0952-7915(93)90012-H 8507401

[32] Guadalupe M Reay E Sankaran S Prindiville T Flamm J Mcneil A Severe CD4+ T-cell depletion in gut lymphoid tissue during primary human immunodeficiency virus type 1 infection and substantial delay in restoration following highly active antiretroviral therapy. J Virol (2003) 77:11708–17. 10.1128/JVI.77.21.11708-11717.2003 14557656 PMC229357

[33] Mehandru S Poles MA Tenner-Racz K Jean-Pierre P Manuelli V Lopez P Lack of mucosal immune reconstitution during prolonged treatment of acute and early HIV-1 infection. PLoS Med (2006) 3:e484. 10.1371/journal.pmed.0030484 17147468 PMC1762085

[34] Rennert PD Hochman PS Flavell RA Chaplin DD Jayaraman S Browning JL Essential role of lymph nodes in contact hypersensitivity revealed in lymphotoxin-alpha-deficient mice. J Exp Med (2001) 193:1227–38. 10.1084/jem.193.11.1227 11390430 PMC2193379

[35] Mehandru S Tenner-Racz K Racz P Markowitz M. The gastrointestinal tract is critical to the pathogenesis of acute HIV-1 infection. J Allergy Clin Immunol (2005) 116:419–22. 10.1016/j.jaci.2005.05.040 16083799

[36] Kim KS. Microbial translocation of the blood-brain barrier. Int J Parasitol (2006) 36:607–14. 10.1016/j.ijpara.2006.01.013 16542662

[37] Redd AD Gray RH Quinn TC Is microbial translocation a cause or consequence of HIV disease progression? J Infect Dis (2011) 203:744–5. 10.1093/infdis/jiq107 21220777 PMC3072727

[38] Smit-McBride Z Mattapallil JJ Mcchesney M Ferrick D Dandekar S. Gastrointestinal T lymphocytes retain high potential for cytokine responses but have severe CD4(+) T-cell depletion at all stages of simian immunodeficiency virus infection compared to peripheral lymphocytes. J Virol (1998) 72:6646–56. 9658111 10.1128/jvi.72.8.6646-6656.1998 PMC109855

[39] Okoye AA Picker LJ. CD4(+) T-cell depletion in HIV infection: mechanisms of immunological failure. Immunol Rev (2013) 254:54–64. 10.1111/imr.12066 23772614 PMC3729334

[40] Giorgi JV Hultin LE Mckeating JA Johnson TD Owens B Jacobson LP Shorter survival in advanced human immunodeficiency virus type 1 infection is more closely associated with T lymphocyte activation than with plasma virus burden or virus chemokine coreceptor usage. J Infect Dis (1999) 179:859–70. 10.1086/314660 10068581

[41] Fox CH Kotler D Tierney A Wilson CS Fauci AS. Detection of HIV-1 RNA in the lamina propria of patients with AIDS and gastrointestinal disease. J Infect Dis (1989) 159:467–71. 10.1093/infdis/159.3.467 2915167

[42] Ehrenpreis ED Ganger DR Kochvar GT Patterson BK Craig RM. d-xylose malabsorption: characteristic finding in patients with the AIDS wasting syndrome and chronic diarrhea. J Acquir Immune Defic Syndr (1992) 5:1047–50. 1453320

[43] Schneider T Ullrich R Bergs C Schmidt W Riecken EO Zeitz M. Abnormalities in subset distribution, activation, and differentiation of T cells isolated from large intestine biopsies in HIV infection. The Berlin Diarrhoea/Wasting Syndrome Study Group. Clin Exp Immunol (1994) 95:430–5. 10.1111/j.1365-2249.1994.tb07014.x 8137540 PMC1535074

[44] Finkel TH Tudor-Williams G Banda NK Cotton MF Curiel T Monks C Apoptosis occurs predominantly in bystander cells and not in productively infected cells of HIV- and SIV-infected lymph nodes. Nat Med (1995) 1:129–34. 10.1038/nm0295-129 7585008

[45] Kinter A Mcnally J Riggin L Jackson R Roby G Fauci AS. Suppression of HIV-specific T cell activity by lymph node CD25+ regulatory T cells from HIV-infected individuals. Proc Natl Acad Sci U S A (2007) 104:3390–5. 10.1073/pnas.0611423104 17360656 PMC1805624

[46] Sage PT Francisco LM Carman CV Sharpe AH. The receptor PD-1 controls follicular regulatory T cells in the lymph nodes and blood. Nat Immunol (2013) 14:152–61. 10.1038/ni.2496 23242415 PMC3788614

[47] Kim JY Ryu MR Choi BO Park WC Oh SJ Won JM The prognostic significance of the lymph node ratio in axillary lymph node positive breast cancer. J Breast Cancer (2011) 14:204–12. 10.4048/jbc.2011.14.3.204 22031802 PMC3200516

[48] Costiniuk CT Angel JB. Human immunodeficiency virus and the gastrointestinal immune system: does highly active antiretroviral therapy restore gut immunity? Mucosal Immunol (2012) 5:596–604. 10.1038/mi.2012.82 22929559

[49] Mowat AM. Anatomical basis of tolerance and immunity to intestinal antigens. Nat Rev Immunol (2003) 3:331–41. 10.1038/nri1057 12669023

[50] Mehandru S The Gastrointestinal Tract in HIV-1 Infection: Questions, Answers, and More Questions! New York, NY: Physicians’ Research Notebook; Physicians’ Research Network Inc (2007).

[51] Veazey R Lackner A. The mucosal immune system and HIV-1 infection. AIDS Rev (2003) 5:245–52. 15012003

[52] Balic A Smith KA Harcus Y Maizels RM. Dynamics of CD11c(+) dendritic cell subsets in lymph nodes draining the site of intestinal nematode infection. Immunol Lett (2009) 127:68–75. 10.1016/j.imlet.2009.09.001 19766674 PMC2789245

[53] Orenstein JM. HIV expression in surgical specimens. AIDS Res Hum Retroviruses (2008) 24:947–55. 10.1089/aid.2008.0265 18671477

[54] Berg RD. Bacterial translocation from the gastrointestinal tract. Trends Microbiol (1995) 3:149–54. 10.1016/S0966-842X(00)88906-4 7613757

[55] Horiike M Iwami S Kodama M Sato A Watanabe Y Yasui M Lymph nodes harbor viral reservoirs that cause rebound of plasma viremia in SIV-infected macaques upon cessation of combined antiretroviral therapy. Virology (2012) 423:107–18. 10.1016/j.virol.2011.11.024 22196013

[56] Cumont MC Monceaux V Viollet L Lay S Parker R Hurtrel B TGF-beta in intestinal lymphoid organs contributes to the death of armed effector CD8 T cells and is associated with the absence of virus containment in rhesus macaques infected with the simian immunodeficiency virus. Cell Death Differ (2007) 14:1747–58. 10.1038/sj.cdd.4402192 17612589

[57] Estaquier J Hurtrel B [Mesenteric lymph nodes, a sanctuary for the persistance of HIV. Escape mechanisms]. Med Sci (Paris) (2008) 24:1055–60. 10.1051/medsci/200824121055 19116114

[58] Okoye A Meier-Schellersheim M Brenchley JM Hagen SI Walker JM Rohankhedkar M Progressive CD4+ central memory T cell decline results in CD4+ effector memory insufficiency and overt disease in chronic SIV infection. J Exp Med (2007) 204:2171–85. 10.1084/jem.20070567082907c 17724130 PMC2118701

