# Supplementary material for: Comparative Analysis of Immune Activation Markers of CD8+ T Cells in Lymph Nodes of Different Origins in SIV-Infected Chinese Rhesus Macaques
Source: Front Immunol. 2016 Sep 21;7:371. doi: 10.3389/fimmu.2016.00371 (PMC5030343; doi:10.3389/fimmu.2016.00371)
Supplement: Supplementary file 1 [file Data_Sheet_1.DOCX]

**Supplementary Figure 1. Correlation analysis of the activation of CD8^+^ T cells with CD4/CD8 ratio in GI LNs (A, B) and peripheral LNs (C, D) of SIV-infected monkeys.**

**Supplementary Figure 2. Peripheral blood T cell activation of chronic SIV-infected macaques.** CD69 and HLA-DR expression on CD4^+^ and CD8^+^ T cells in the blood of uninfected and SIV chronic infected macaques. N represents the number of animals in each group.
